# Development of muscle weakness in a mouse model of critical illness: does fibroblast growth factor 21 play a role?

**DOI:** 10.1186/s13395-023-00320-4

**Published:** 2023-08-04

**Authors:** Wouter Vankrunkelsven, Steven Thiessen, Sarah Derde, Ellen Vervoort, Inge Derese, Isabel Pintelon, Hanne Matheussen, Alexander Jans, Chloë Goossens, Lies Langouche, Greet Van den Berghe, Ilse Vanhorebeek

**Affiliations:** 1 Clinical Division and Laboratory of Intensive Care Medicine, Department of Cellular and Molecular Medicine, KU Leuven, Herestraat 49, B-3000 Leuven, Belgium; 2 Laboratory of Cell Biology and Histology, University of Antwerp, Antwerp, Belgium

**Keywords:** FGF21, Critical illness, Muscle mass, Muscle weakness, Mitochondrial function, Endoplasmic reticulum stress, Autophagy

## Abstract

**Background:**

Critical illness is hallmarked by severe stress and organ damage. Fibroblast growth factor 21 (FGF21) has been shown to rise during critical illness. FGF21 is a pleiotropic hormone that mediates adaptive responses to tissue injury and repair in various chronic pathological conditions. Animal studies have suggested that the critical illness-induced rise in FGF21 may to a certain extent protect against acute lung, liver, kidney and brain injury. However, FGF21 has also been shown to mediate fasting-induced loss of muscle mass and force. Such loss of muscle mass and force is a frequent problem of critically ill patients, associated with adverse outcome. In the present study, we therefore investigated whether the critical illness-induced acute rise in FGF21 is muscle-protective or rather contributes to the pathophysiology of critical illness-induced muscle weakness.

**Methods:**

In a catheterised mouse model of critical illness induced by surgery and sepsis, we first assessed the effects of genetic FGF21 inactivation, and hence the inability to acutely increase FGF21, on survival, body weight, muscle wasting and weakness, and markers of muscle cellular stress and dysfunction in acute (30 h) and prolonged (5 days) critical illness. Secondly, we assessed whether any effects were mirrored by supplementing an FGF21 analogue (LY2405319) in prolonged critical illness.

**Results:**

FGF21 was not required for survival of sepsis. Genetic FGF21 inactivation aggravated the critical illness-induced body weight loss (*p* = 0.0003), loss of muscle force (*p* = 0.03) and shift to smaller myofibers. This was accompanied by a more pronounced rise in markers of endoplasmic reticulum stress in muscle, without effects on impairments in mitochondrial respiratory chain enzyme activities or autophagy activation. Supplementing critically ill mice with LY2405319 did not affect survival, muscle force or weight, or markers of muscle cellular stress/dysfunction.

**Conclusions:**

Endogenous FGF21 is not required for sepsis survival, but may partially protect muscle force and may reduce cellular stress in muscle. Exogenous FGF21 supplementation failed to improve muscle force or cellular stress, not supporting the clinical applicability of FGF21 supplementation to protect against muscle weakness during critical illness.

**Supplementary Information:**

The online version contains supplementary material available at 10.1186/s13395-023-00320-4.

## Background

Critically ill patients often develop muscle weakness during their stay in the intensive care unit (ICU), apart from failure of multiple other vital organ systems [1–3]. The pathophysiology remains incompletely understood. Both polyneuropathy and myopathy may contribute to ICU-acquired weakness, with myopathic changes including a decrease in muscle mass and quality [1, 4]. An imbalance between protein synthesis and breakdown contributes to the loss of muscle mass. Cellular dysfunction, hallmarked by mitochondrial dysfunction, insufficient autophagy activation and endoplasmic reticulum (ER) stress, contributes to the loss of muscle quality and function [1, 4, 5].

Fibroblast growth factor 21 (FGF21) is a pleiotropic hormone that regulates glucose and lipid metabolism, amongst other functions, and is induced in response to various metabolic and cellular stresses [6]. Whereas the liver is the main source of circulating FGF21 under physiological circumstances, FGF21 can also be induced in and secreted from other tissues into the bloodstream upon cellular stress [7]. In this regard, mitochondrial dysfunction, impaired autophagy, and ER stress can trigger muscle and hepatic expression and secretion of FGF21 [9–11]. Consequently, FGF21 serum concentrations are elevated in several diseases hallmarked by mitochondrial dysfunction and ER stress, such as obesity, diabetes and mitochondrial myopathies, regarded as an adaptive response by increasing mitochondrial respiration and autophagy, and attenuating ER stress and metabolic abnormalities [8, 10, 12–14]. However, this positive view has been challenged, as some detrimental effects have been attributed to FGF21 that may be responsible for disease progression [15]. In humans, FGF21 appeared to be a predictor for disease progression in several chronic conditions, like end-stage renal disease and diastolic heart failure, and to correlate with mortality in the elderly [15–17]. In mice, FGF21 has been shown to contribute to fasting-induced muscle atrophy and to a pro-senescence metabolic shift [18, 19].

Interestingly, serum FGF21 concentrations acutely increase in critically ill patients upon ICU admission, gradually decrease, but remain elevated at ICU discharge [20, 21], even more so in non-survivors than in survivors. Studies in animal models of sepsis-induced critical illness suggested that FGF21 deficiency increased mortality, whereas FGF21 supplementation may decrease mortality [22, 23]. A protective role of FGF21 has also been suggested in the context of critical insults on the lung, liver, kidney and brain [24–31]. However, the effect of FGF21 on muscle during critical illness remained to be investigated, where theoretically the rise in FGF21 during critical illness may reflect an adaptive, beneficial response aimed at restoring muscle homeostasis, or may rather contribute to the pathophysiology of critical illness-induced muscle weakness. Therefore, we studied the impact of both loss of FGF21 and supplementation with an FGF21 analogue on muscle in a mouse model of critical illness induced by surgery and sepsis.

## Methods

### Animal study design

We performed 2 studies in a mouse model of critical illness induced by surgery and sepsis. For this mouse model, we used only male mice to avoid the cyclic influence of estrogens [32]. After anaesthesia (intraperitoneal injection of ketamine:xylazine (100:13 mg/kg), a catheter was placed in the central jugular vein, followed by caecal ligation (50% of its length) and a through-and-through puncture with an 18G needle to induce polymicrobial sepsis (CLP) [32, 33]. After surgery, mice were transferred to individual cages. They were fasted and received intravenous fluid resuscitation with balanced colloids and crystalloids (1:4 ratio; 0.3 ml/hour), to mimic the clinical setting in which parenteral nutrition (PN) is initially withheld. In studies lasting longer than 30 h, mice received standard mixed PN (0.2 ml/hour; 5.8 kcal/day; Olimel N7E, Baxter, Lessines, Belgium) starting 24 h after surgery. Throughout the study, starting 6 h after CLP, mice received twice daily subcutaneous injections with antibiotics (Imipenem/Cilastin; 16.67 mg/kg) and analgesics (Buprenorphine; 0.15 mg/kg). Pain/discomfort was assessed twice daily [34]. Individually caged healthy mice receiving standard chow at the same daily caloric intake as critically ill mice (pair-fed) were used as controls. As we aimed to study the impact of critical illness rather than the separate impact of the sepsis component, considering that surgery and sepsis often co-occur in critically ill patients, we did not include a surgical control group.

In a first study, we used 16-weeks old *Fgf21*^−/−^ and *Fgf21*^+*/*+^ male mice (breeding pairs kindly provided by Profs. Kliewer and Mangelsdorf, UT Southwestern Medical Center, Dallas, TX [35]). Mice were sacrificed 30 h or 125 h (5 days) post-CLP, representing the acute and prolonged phase of critical illness. The 5-days time point was chosen to allow development of a clear phenotype of muscle weakness in this model [36, 37]. At sacrifice, the animals were deeply anaesthetised, blood was drawn via cardiac puncture and organs were harvested. In a second study, we administered the FGF21 analogue LY2405319 (kindly provided by Dr. Benjamin Yaden, Eli Lilly, Indianapolis, IN) [38] twice daily in a total daily dose of 5 mg/kg or placebo to 24-weeks old male C57BL/6 J mice (Janvier, Le Genest-Saint-Isle, France), starting 10 h after CLP. Mice were sacrificed 125 h (5 days) post-CLP. The studies were continued until at least 15 surviving animals (30 h study) or at least 15 surviving animals with successful muscle force measurements had been included per group (allowing demonstration of a 25% increase in peak tetanic force with 80% power and 95% certainty). The total daily dose of LY2405319 at 5 mg/kg/day was selected based on previous studies [13]. To verify biological activity of the compound, we evaluated the impact of a 5-days treatment of healthy mice (pair-fed to the critically ill mice) with this dosing regime versus placebo on plasma triglycerides, free glycerol and free fatty acids.

A randomised complete block design was used for group allocation. Staff performing mouse studies and tissue harvesting were not blinded for healthy control versus critically ill mice, but were blinded for *Fgf21* genotype or placebo versus LY2405319 administration. All laboratory measurements were performed completely blinded for group allocation.

The KU Leuven Institutional Ethical Committee for Animal Experimentation approved all studies and protocols (P093/2014). Animals were treated according to the US National Institutes of Health “Guide for the Care and Use of Laboratory Animals”. All experiments complied with the Animal Research-Reporting In Vivo Experiment (ARRIVE) guidelines.

### Ex vivo muscle force measurements

In mice that survived 125 h, the hindlimb m. extensor digitorum longus (EDL) was carefully dissected directly after euthanasia and suspended in a temperature-controlled (30 °C) organ bath filled with HEPES-fortified Krebs–Ringer solution to measure muscle force (300C-LR Dual-Mode muscle lever, Aurora Scientific, Ontario, Canada) [36]. The small size of the EDL guaranteed proper diffusion of oxygen during the procedure. Maximal isometric tetanic force was measured by averaging three consecutive tetanic stimuli (180 Hz stimulation frequency, 200 ms duration, 0.2 ms pulse width, 2 min rest intervals).

### MRI whole body composition analyses

In the second study, body composition was measured immediately before surgery and immediately before sacrifice for critically ill mice, and at equivalent time points for healthy control mice, with use of magnetic resonance imaging (echoMRI-100H, Whole Body Magnetic Resonance Analyser, Zinsser Analytic GmbH, Germany).

### Whole blood and plasma analyses

Plasma concentrations of human (exogenous, LY2405319) and mouse (endogenous) FGF21 were measured with ELISA (Human FGF21 Quantikine ELISA and Mouse/Rat Quantikine ELISA, R&D Systems, Abingdon, UK). Free fatty acids (FFAs, Cayman Chem, Ann Arbor, MI), glycerol (Sigma-Aldrich, Saint-Louis, MO), and triglycerides (Abcam, Cambridge, UK) were measured with fluorimetric or colorimetric assays.

### Tissue analyses

RNA isolation, reverse transcription, and real-time polymerase chain reaction for gene expression analyses in gastrocnemius muscle, corresponding commercial assays, and normalisation to the housekeeping gene 18S ribosomal RNA are described in Additional file 1 (Methods S1, Table S1, Table S2). Protein isolation from gastrocnemius muscle, immunoblotting, the corresponding antibodies and visualisation and quantification are described in Additional file 1 (Methods S2 and Table S3). Citrate synthase and mitochondrial respiratory chain complex activities in tibialis anterior muscle were measured with spectrophotometry at 30 °C [39], with details provided in Additional file 1 (Methods S3). Histological analyses for quantification of myofiber size distribution and evaluation of general morphology in tibialis anterior muscle are described in Additional file 1 (Methods S4).

### Statistical analysis

Two-way-ANOVA assessed differences among genotypes and among healthy and ill mice, as well as interaction between genotype and illness, if necessary after transformation to obtain a near-normal data distribution. When significance was obtained, groups were further compared 2 by 2 based on non-transformed data, with t-tests (normally distributed data) or non-parametric Wilcoxon rank-sum tests (not-normally distributed data). Two-way-ANOVA results are only given for measures already showing a significant difference among genotypes under healthy pair-fed condition, with reporting of the corresponding interaction p-value. Survival was visualised with Kaplan–Meier curves and compared among groups with the log-rank test. Two-sided p-values below 0.05 were considered statistically significant. Data are presented as box plots showing medians with interquartile ranges (IQR), and whiskers drawn to the furthest point within 1.5 times the IQR from the box. Statistical analyses were performed with JMP Pro 15 or JMP Pro 17.0.0 (SAS Institute Inc, Cary, NC).

## Results

### Impact of critical illness on FGF21 and of loss of FGF21 on mortality and body weight

**Fig. 1:**
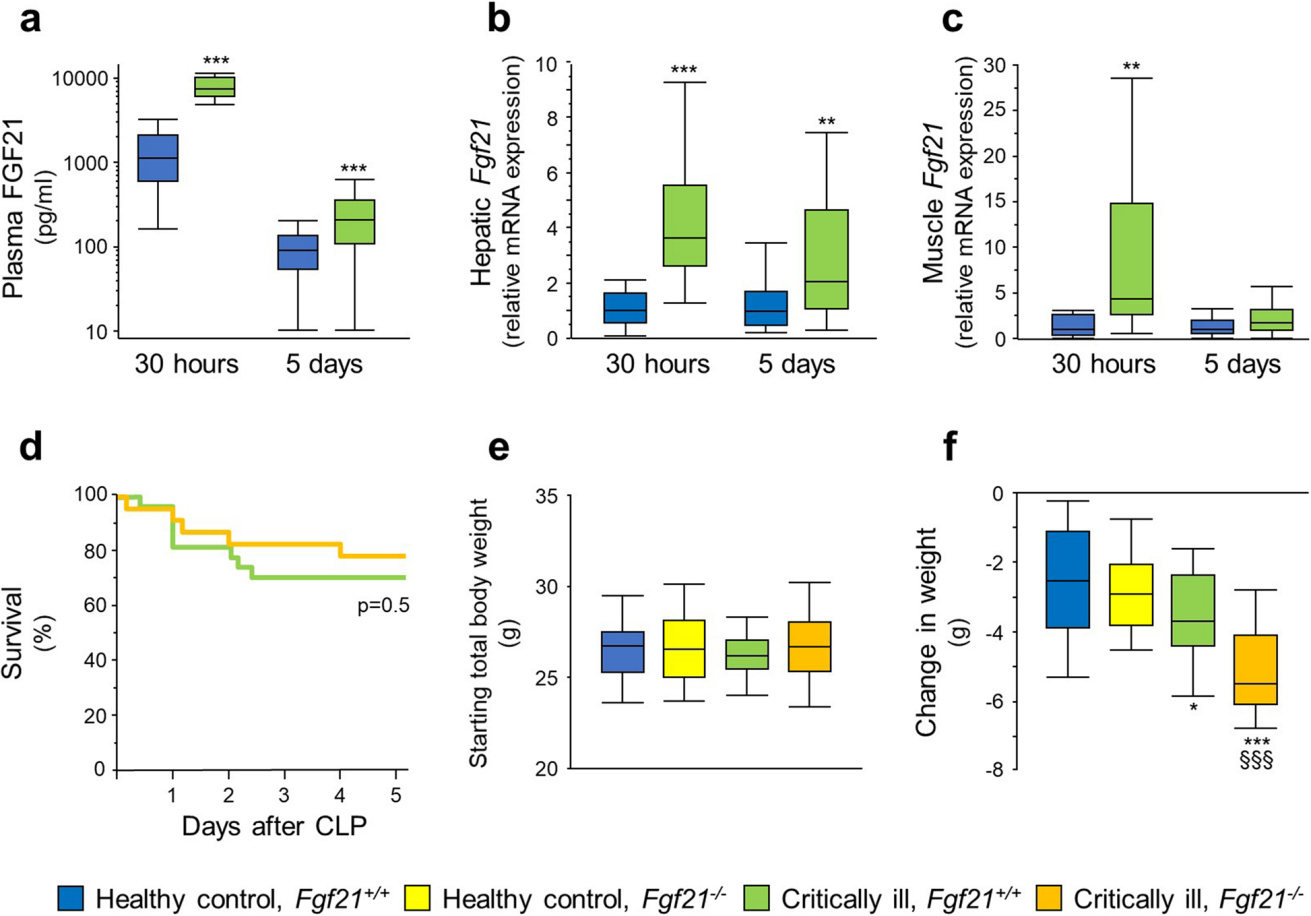
Impact of critical illness on FGF21, and of FGF21-loss on critical illness-induced mortality and body-weight loss. **a** Plasma FGF21 concentrations in healthy and critically ill *Fgf21*^+/+^ mice. **b-c** Relative mRNA expression of *Fgf21* in liver and gastrocnemius muscle of healthy and critically ill *Fgf21*^+/+^ mice. Gene expression data are shown relative to the mean of healthy *Fgf21*^+*/*+^ mice at each time point. **d** Survival of critically ill *Fgf21*^+/+^ and *Fgf21*^−/−^ mice. In the acute phase study (not shown), 2 of 17 (11.8%) *Fgf21*^+/+^ mice and 2 of 17 (11.8%) *Fgf21*^−/−^ mice died within 30 h after CLP (p > 0.99). In the 5 days study, 8 of 27 (29.6%) *Fgf21*^+/+^ mice and 5 of 23 (21.7%) *Fgf21*^−/−^ mice did not survive the preset time period of critical illness (Logrank *p* = 0.5). **e** Starting total body weight of mice included in the 30 h and the 5 days study. **f** Change in body weight after 5 days of critical illness or pair-feeding in *Fgf21*^+/+^ and *Fgf21*^−/−^ mice as compared to baseline. **a-c**, **f** Healthy control, *Fgf21*^+*/*+^: *n* = 15 at 30 h, *n* = 21 at 5 days; Healthy control, *Fgf21*^*−/−*^: *n* = 15 at 30 h, *n* = 24 at 5 days; Sepsis, *Fgf21*^+*/*+^: *n* = 15 at 30 h, *n* = 19 at 5 days; Sepsis, *Fgf21*^*−/−*^: *n* = 15 at 30 h, *n* = 18 at 5 days. **e** Healthy control, *Fgf21*^+*/*+^: *n* = 36; Healthy control, *Fgf21*^*−/−*^: *n* = 39; Critically ill, *Fgf21*^+*/*+^: *n* = 44; Critically ill, *Fgf21*^*−/−*^: *n* = 40. * *p* < 0.05, ** *p* < 0.01, *** *p* < 0.001 between healthy control and critically ill mice and §§§ *p* < 0.001 between *Fgf21*^+/+^ and *Fgf21*^−/−^ mice

We first assessed the impact of critical illness versus pair-feeding (i.e. fasting in the acute phase, 45% of caloric needs from day 2 onwards) on FGF21 in *Fgf21*^+*/*+^ mice. We therefore measured plasma concentrations, gene expression in liver as the main source of circulating FGF21 under physiological circumstances, and gene expression in muscle as the tissue of interest. Thirty hours after CLP, plasma FGF21 concentrations were 5.7-fold higher than in pair-fed healthy mice (*p* < 0.0001, Fig. 1a). FGF21 decreased in both groups by day 5, but was still 3.3-fold higher in ill than in pair-fed healthy mice (*p* = 0.0002). Critical illness also increased *Fgf21* gene expression in liver at both time points (3.6- and 2.4-fold) and in muscle after 30 h (5.6-fold) (*p* ≤ 0.009, Fig. 1b-c). In *Fgf21*^*−/−*^ mice, plasma FGF21 and *Fgf21* gene expression in liver and muscle were undetectable (Fig. 1a-c), confirming *Fgf21* inactivation (Additional file 1, Fig. S1). Loss of FGF21 did not affect 5-day survival of critically ill mice (Fig. 1d). Total body weight at baseline was comparable among all groups (Fig. 1e). After 5 days, both critically ill *Fgf21*^+*/*+^ (*p* = 0.02) and *Fgf21*^*−/−*^ (*p* < 0.0001) mice had lost more weight than the corresponding pair-fed healthy mice (Fig. 1f). However, critically ill *Fgf21*^*−/−*^ mice had lost more weight than critically ill *Fgf21*^+*/*+^ mice (*p* = 0.0003).

### Effect of loss of FGF21 on muscle wasting and weakness during critical illness

**Fig. 2:**
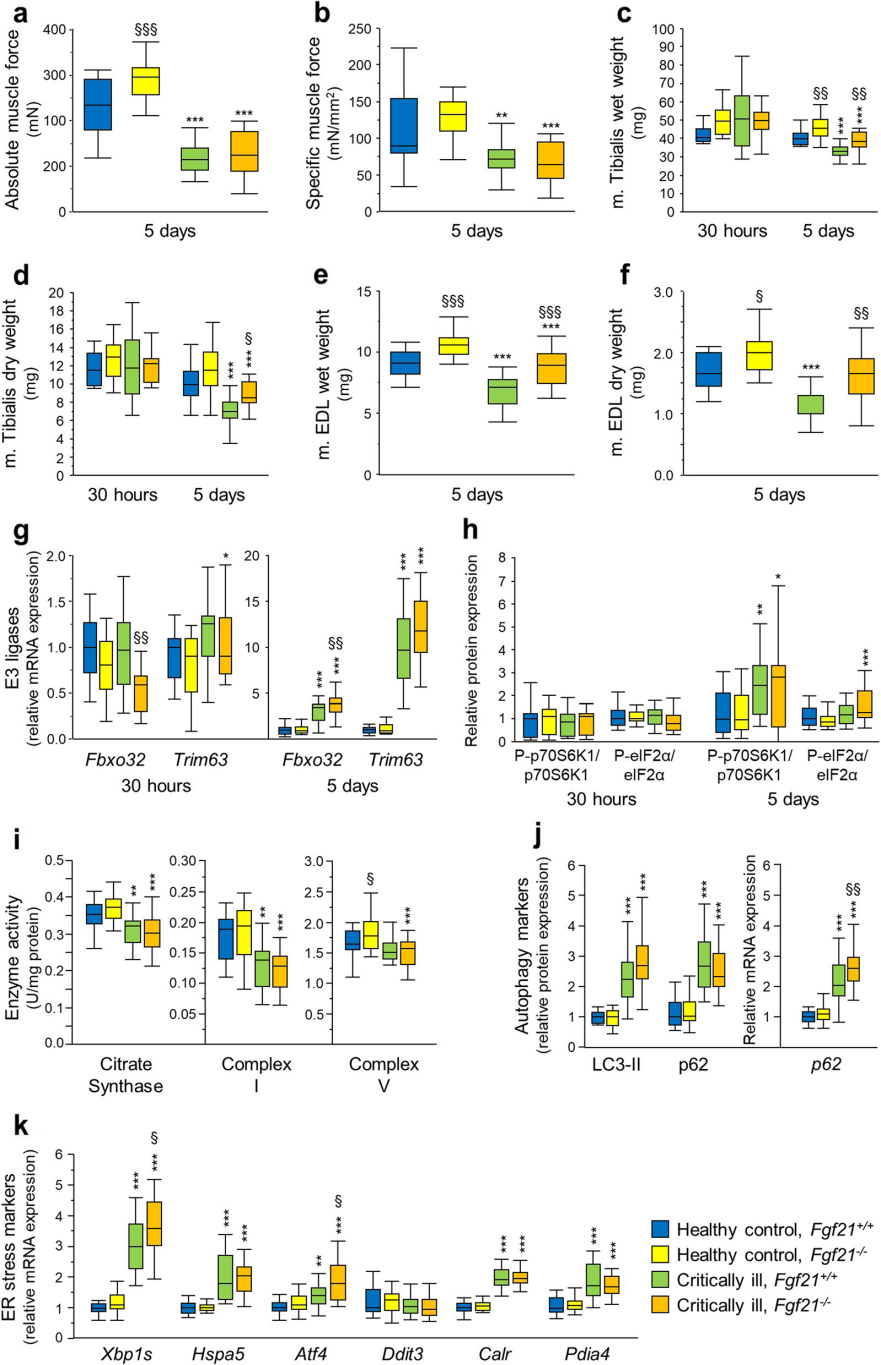
Effect of loss of FGF21 on critical illness-induced muscle wasting and weakness. **a** EDL muscle absolute maximal tetanic force. 2-way-ANOVA interaction *p* = 0.03. **b** EDL muscle specific maximal tetanic force. **c** Tibialis muscle wet weight. At 5 days, 2-way-ANOVA *p* = 0.6. **d** Tibialis muscle dry weight. **e** EDL muscle wet weight. 2-way-ANOVA *p* = 0.5. **f** EDL muscle dry weight. 2-way-ANOVA *p* = 0.5. **g** Muscle atrophy markers. Relative mRNA expression of F-box protein 32 (also known as Atrogin-1) (*Fbxo32*) and Tripartite motif containing 63 (also known as Muscle-ring-finger-1) (*Trim63*) in gastrocnemius muscle. **h** Ratio of phosphorylated over total p70S6 kinase (p70S6K1) and Eukaryotic translation initiation factor 2α (eIF2α) protein expression in gastrocnemius muscle. **i** Markers of mitochondrial function. Muscle citrate synthase and respiratory chain complex I and V activity in tibialis anterior muscle. Complex V 2-way-ANOVA *p* = 0.2. **j** Autophagy markers. Relative protein expression of Microtubule-associated protein light chain-3, mature-form (LC3-II) and p62 and relative mRNA expression of *p62* in gastrocnemius muscle. **k** Relative mRNA expression of ER stress response markers, Spliced X-box binding protein 1 (*Xbp1s*), Heat shock protein family A member 5 (*Hspa5*), Activating transcription factor 4 (*Atf4*), DNA damage inducible transcript 3 (*Ddit3*), Calreticulin (*Calr*), Protein disulphide isomerase family A member 4 (*Pdia4*) in gastrocnemius muscle. Gene and protein expression are shown relative to the mean of healthy *Fgf21*^+*/*+^ mice. **a**, **b**, **e**, **f** Healthy control, *Fgf21*^+*/*+^: *n* = 15; Healthy control, *Fgf21*^*−/−*^: *n* = 16; Critically ill, *Fgf21*^+*/*+^: *n* = 14; Critically ill *Fgf21*^*−/−*^: *n* = 16. **c**, **d**, **g**, **h** Healthy control, *Fgf21*^+*/*+^: *n* = 15 at 30 h, *n* = 21 at 5 days; Healthy control, *Fgf21*^*−/−*^: *n* = 15 at 30 h, *n* = 24 at 5 days; Critically ill, *Fgf21*^+*/*+^: *n* = 15 at 30 h, *n* = 19 at 5 days; Critically ill, *Fgf21*^*−/−*^: *n* = 15 at 30 h, *n* = 18 at 5 days. **i-k** Healthy control, *Fgf21*^+*/*+^: *n* = 21; Healthy control, *Fgf21*^*−/−*^: *n* = 24; Critically ill, *Fgf21*^+*/*+^: *n* = 19; Critically ill, *Fgf21*^*−/−*^: *n* = 18. * *p* < 0.05, ** *p* < 0.01, *** *p* < 0.001 between healthy control and critically ill mice and § *p* < 0.05, §§ *p* < 0.01 and §§§ *p* < 0.001 between *Fgf21*^+/+^ and *Fgf21*^−/−^ mice

Peak absolute tetanic muscle force of the m. EDL was higher in healthy *Fgf21*^*−/−*^ as compared with *Fgf21*^+*/*+^ mice after 5 days of pair-feeding (*p* = 0.0004, Fig. 2a). Critical illness reduced force in both groups (*p* < 0.0001). However, this reduction was more pronounced in *Fgf21*^*−/−*^ than in *Fgf21*^+*/*+^ mice (interaction *p* = 0.03), which resulted in a comparable force for both ill groups. Impact of FGF21 was less clear when focusing on specific muscle force (normalised to muscle cross section) with only a trend for interaction between genotype and illness (interaction *p* = 0.09, Fig. 2b).

**Fig. 3:**
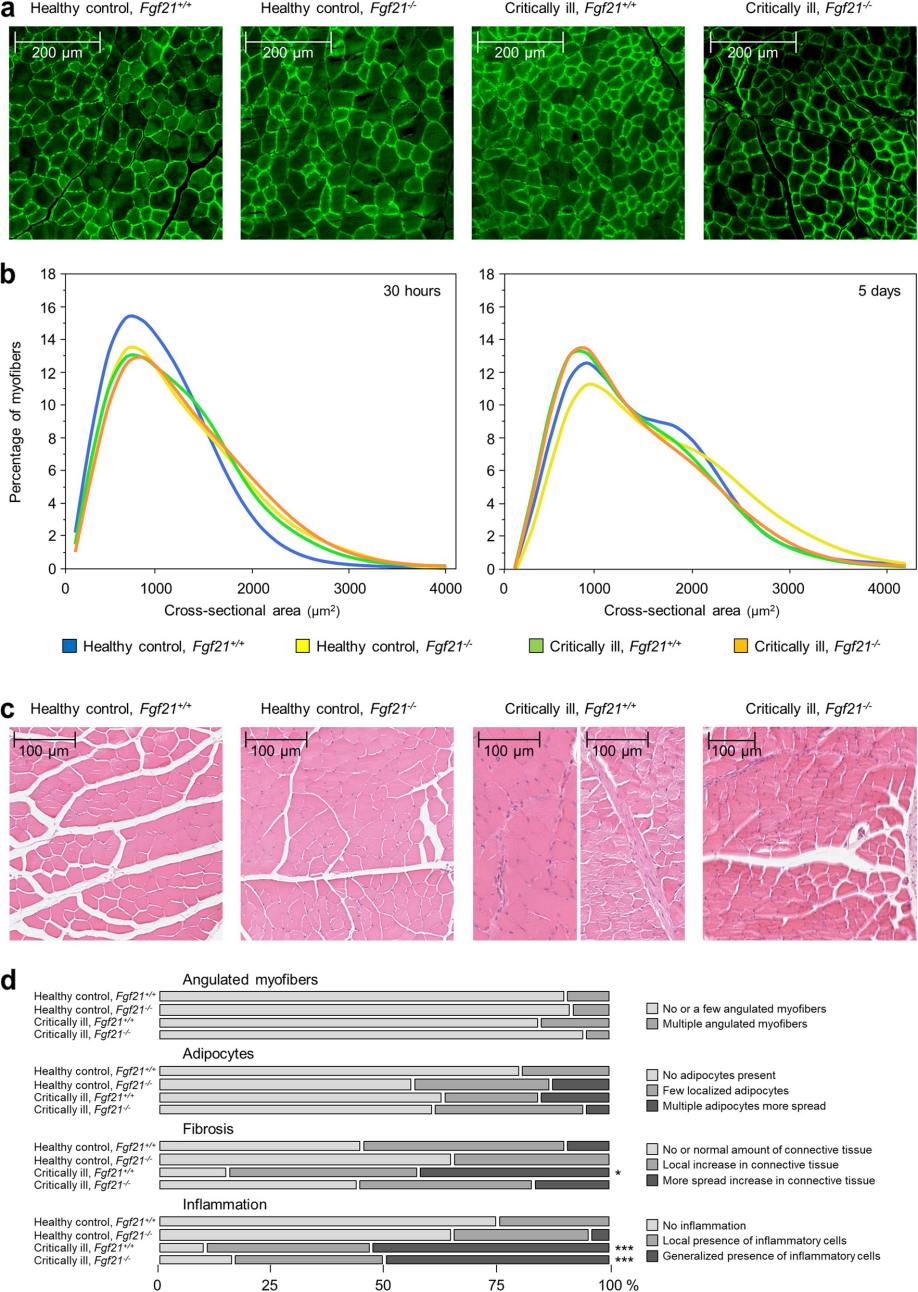
Effect of critical illness and loss of FGF21 on muscle histology. **a** Tibialis anterior sections immunostained for laminin. **b** Tibialis anterior myofiber size distribution. **c** Illustration of tibialis anterior general histology after hematoxylin–eosin staining. **d** Semi-quantitative scoring of tibialis anterior histology. Data are shown as cumulative percentages of the respective group. Healthy control, *Fgf21*^+*/*+^: *n* = 11 at 30 h, *n* = 20 at 5 days; Healthy control, *Fgf21*^*−/−*^: *n* = 11 at 30 h, *n* = 23at 5 days; Critically ill, *Fgf21*^+*/*+^: *n* = 15 at 30 h, *n* = 19 at 5 days; Critically ill, *Fgf21*^*−/−*^: *n* = 15 at 30 h, *n* = 18 at 5 days. * *p* < 0.05, *** *p* < 0.001 between healthy control and critically ill mice

At the 30 h time point, muscle wet and dry weight were comparable for all groups. After 5 days of pair-feeding, m. tibialis anterior wet weight was higher in *Fgf21*^*−/−*^ than in *Fgf21*^+*/*+^ healthy mice (*p* = 0.007, Fig. 2c). However, 5 days of illness reduced muscle mass to a similar extent in *Fgf21*^+*/*+^ (*p* < 0.0001) and *Fgf21*^*−/−*^ (*p* = 0.0004) mice as compared with healthy pair-fed mice, resulting in a higher residual muscle mass in *Fgf21*^*−/−*^ than in *Fgf21*^+*/*+^ critically ill mice (*p* = 0.002). Similar results were obtained for tibialis anterior dry weight and for EDL wet and dry weight (Fig. 2d-f). Healthy *Fgf21*^*−/−*^ mice showed more larger myofibers in tibialis anterior muscle than healthy *Fgf21*^+*/*+^ mice at 30 h and after 5 days of pair-feeding (Fig. 3a,b). A critical illness-induced shift towards smaller myofibers was only observed after 5 days and was more pronounced for *Fgf21*^*−/−*^ than for *Fgf21*^+*/*+^ mice, resulting in a comparable myofiber size distribution for both critically ill groups. After 5 days, muscle of critically ill mice showed a more pronounced presence of connective tissue and of inflammatory cells than healthy pair-fed mice, to a similar extent in *Fgf21*^+*/*+^ and *Fgf21*^+*/*+^ mice (Fig. 3c,d). We then investigated whether an effect on protein breakdown or synthesis could explain the higher muscle mass in *Fgf21*^*−/−*^ mice. With regard to atrophy markers of the ubiquitin–proteasome system, loss of FGF21 decreased mRNA expression of *Fbxo32* (*p* = 0.003) after 30 h in critically ill as compared with healthy pair-fed mice, but slightly increased the critical illness-induced rise in *Fbxo32* after 5 days (*p* = 0.006), and did not significantly affect *Trim63* expression (Fig. 2g). Phosphorylation of p70S6K, downstream of mammalian target of rapamycin (mTOR) as regulator of protein synthesis, was not affected after 30 h of illness, but increased in critically ill mice as compared with pair-fed healthy mice after 5 days of illness, similarly in *Fgf21*^+*/*+^ (*p* = 0.001) and *Fgf21*^*−/−*^ (*p* = 0.01) mice (Fig. 2h, Additional file 1 Fig. S2). Phosphorylation of eIF2α, another regulator of protein synthesis, was not affected by critical illness in *Fgf21*^+*/*+^ mice but increased in *Fgf21*^*−/−*^ mice, after 5 days of illness only (*p* < 0.0001, Fig. 2h, Additional file 1 Fig. S2).

Next, we evaluated whether pathways of cellular dysfunction could have affected muscle force, focusing on the 5 days post-CLP time point where a clear muscle phenotype developed [36, 37]. In healthy pair-fed mice, loss of FGF21 did not affect the activity of the *mitochondrial enzymes* citrate synthase and respiratory chain enzyme complex I, but increased complex V activity (*p* = 0.02, Fig. 2i). After 5 days of illness, the mitochondrial enzyme activities decreased, without impact of loss of FGF21. The effect of critical illness on complex I and V activity was less pronounced or disappeared when expressed as citrate synthase ratio (Additional file 1 Fig. S3). Regarding *autophagy*, critical illness increased the LC3-II protein as marker of autophagosome formation and mRNA and protein expression of the autophagy substrate p62 (*p* < 0.0001, Fig. 2j, Additional file 1 Fig. S2). Responses were similar in *Fgf21*^+*/*+^ and *Fgf21*^*−/−*^ mice, except for a more pronounced increase in *p62* mRNA in critically ill *Fgf21*^*−/−*^ than in *Fgf21*^+*/*+^mice (*p* = 0.007). Critical illness induced *ER stress* in *Fgf21*^+*/*+^ and *Fgf21*^*−/−*^ mice as shown by increases in markers of the unfolded protein response (UPR) to ER stress (Fig. 2h,k). This included the Inositol-requiring enzyme 1α (IRE1α) (*Xbp1s* and *Hspa5*), PKR-like endoplasmic reticulum kinase (PERK) (p-eIF2α/eIF2α ratio, *Aft4* and *Ddit3*) and Activating transcription factor 6 (ATF6) (*Calr* and *Pdia4*) branches of the UPR. In *Fgf21*^*−/−*^ mice, a more pronounced effect was observed on the former 2 as compared with *Fgf21*^+*/*+^ mice.

### Effect of FGF21 supplementation on muscle wasting and weakness during critical illness

Since FGF21 may play a role in the regulation of muscle mass and force, we investigated the effects of a 5 days supplementation with a human FGF21 analogue (LY2405319) versus placebo during critical illness. We first assessed biological activity of the LY2405319 stock in healthy mice, where such supplementation is expected to decrease plasma triglycerides, free glycerol and free fatty acids. The 5 days LY2405319 supplementation, which resulted in plasma LY2405319 concentrations of 80 (11–127) ng/ml, indeed decreased plasma triglycerides from 35 (22–43) to 11 (7–16) mg/dl (*p* < 0.0001), free glycerol from 282 (237–355) to 161 (107–223) µmol/l (*p* = 0.0028) and free fatty acids from 125 (87–173) to 87 (48–116) µmol/l (*p* = 0.036), confirming biological activity.

**Fig. 4:**
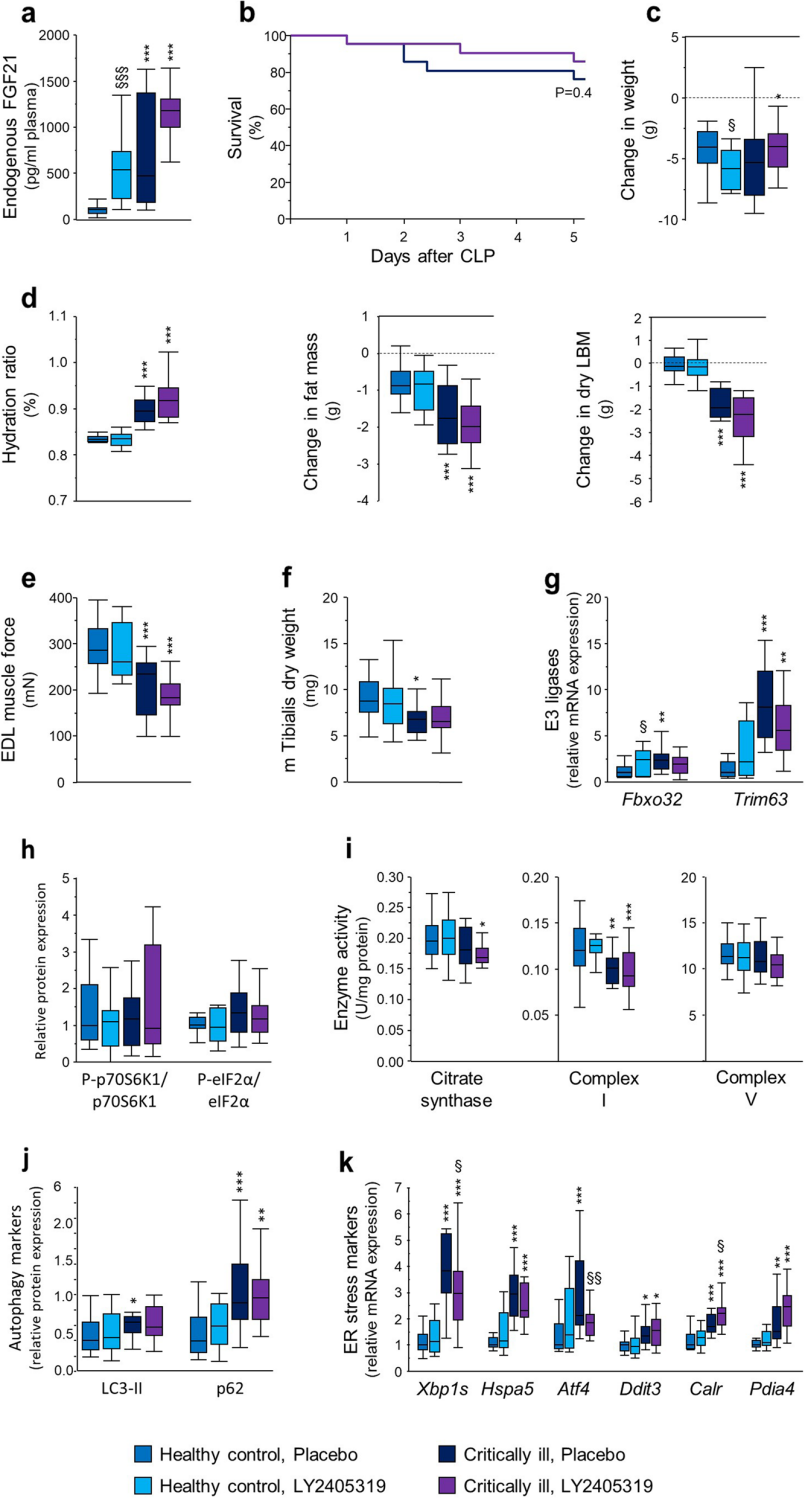
Effect of LY2405319 supplementation on muscle during critical illness. **a** Plasma FGF21 concentrations in healthy mice and placebo- or LY2405319-treated critically ill mice. **b** Survival of critically ill mice treated with placebo or LY2405319, 5 of 21 (23.8%) placebo-treated mice and 3 of 21 (14.3%) LY2405319-treated mice did not survive the preset time period of critical illness (Logrank *p* = 0.4). **c** Change in body weight after 5 days of critical illness or pair-feeding. **d** Hydration ratio, fat mass loss and dry lean body mass (LBM) loss, quantified based on MRI measurements. **e** Absolute maximal tetanic force of the EDL muscle as measured ex vivo. **f** Musculus tibialis anterior dry weight. **g** Relative mRNA expression of markers of atrophy in gastrocnemius muscle. **h** Ratio of phosphorylated over total p70S6K1 and eIF2α protein expression in gastrocnemius muscle. **i** Markers of mitochondrial function in tibialis anterior muscle. Citrate synthase, complex I and V activity. **j** Markers of autophagy in gastrocnemius muscle. Relative protein expression of LC3-II and p62. **k** Relative mRNA expression of markers of ER stress in gastrocnemius muscle. Gene expression data are shown relative to the mean of healthy pair-fed mice. **a**, **c**, **d**, **f-k** Healthy control, placebo: *n* = 18; Healthy control, LY2405319: *n* = 16; Critically ill, placebo: *n* = 16; Critically ill, LY2405319: *n* = 18. **e** Healthy control: *n* = 15; Healthy control, LY2405319: *n* = 14; Critically ill, placebo: *n* = 15; Critically ill, LY2405319: *n* = 15. * *p* < 0.05, ** *p* < 0.01, *** *p* < 0.001 between healthy control and critically ill mice and § *p* < 0.05, §§ *p* < 0.01 between mice treated with placebo or with LY2405319

Endogenous mouse FGF21 plasma concentrations (LY2405319 is not picked up by this assay) increased in both critically ill groups versus pair-fed healthy mice (*p* < 0.0001, Fig. 4a). LY2405319 plasma concentrations were 161 (92–239) in critically ill mice. LY2405319 supplementation during critical illness did not affect survival (Fig. 4b). LY2405319 supplementation aggravated weight loss in pair-fed healthy mice versus placebo, but weight loss was comparable for placebo- and LY2405319-treated critically ill mice (Fig. 4c). LY2405319 did not affect critical illness-induced oedema (evidenced by an increased hydration ratio on MRI), or loss of fat mass or dry (oedema-corrected) lean body mass (Fig. 4d).

LY2405319 did not affect critical illness-induced loss of muscle force (Fig. 4e). Whereas critical illness reduced muscle weight under placebo, this effect failed to reach statistical significance in LY245319-treated mice (Fig. 4f). LY245319 increased gene expression of *Fbxo32* in healthy mice as such that no further rise was seen in LY245319-treated critically ill mice, unlike in placebo-treated mice (Fig. 4g). LY245319 did not affect gene expression of *Trim63* (Fig. 4g), phosphorylation of p70S6K1 or eIF2α (Fig. 4h), mitochondrial enzyme activities (Fig. 4i, Additional file 1 Fig. S4) or markers of autophagy (Fig. 4j). Concerning ER stress in muscle, LY2405319 attenuated the rise of *Xbp1s* (*p* = 0.02, IRE1α branch) and *Atf4* (*p* = 0.009, PERK branch) but increased *Calr* (*p* = 0.01, ATF6 branch, Fig. 4k).

### FGF21 receptor expression in muscle of critically ill mice

**Fig. 5:**
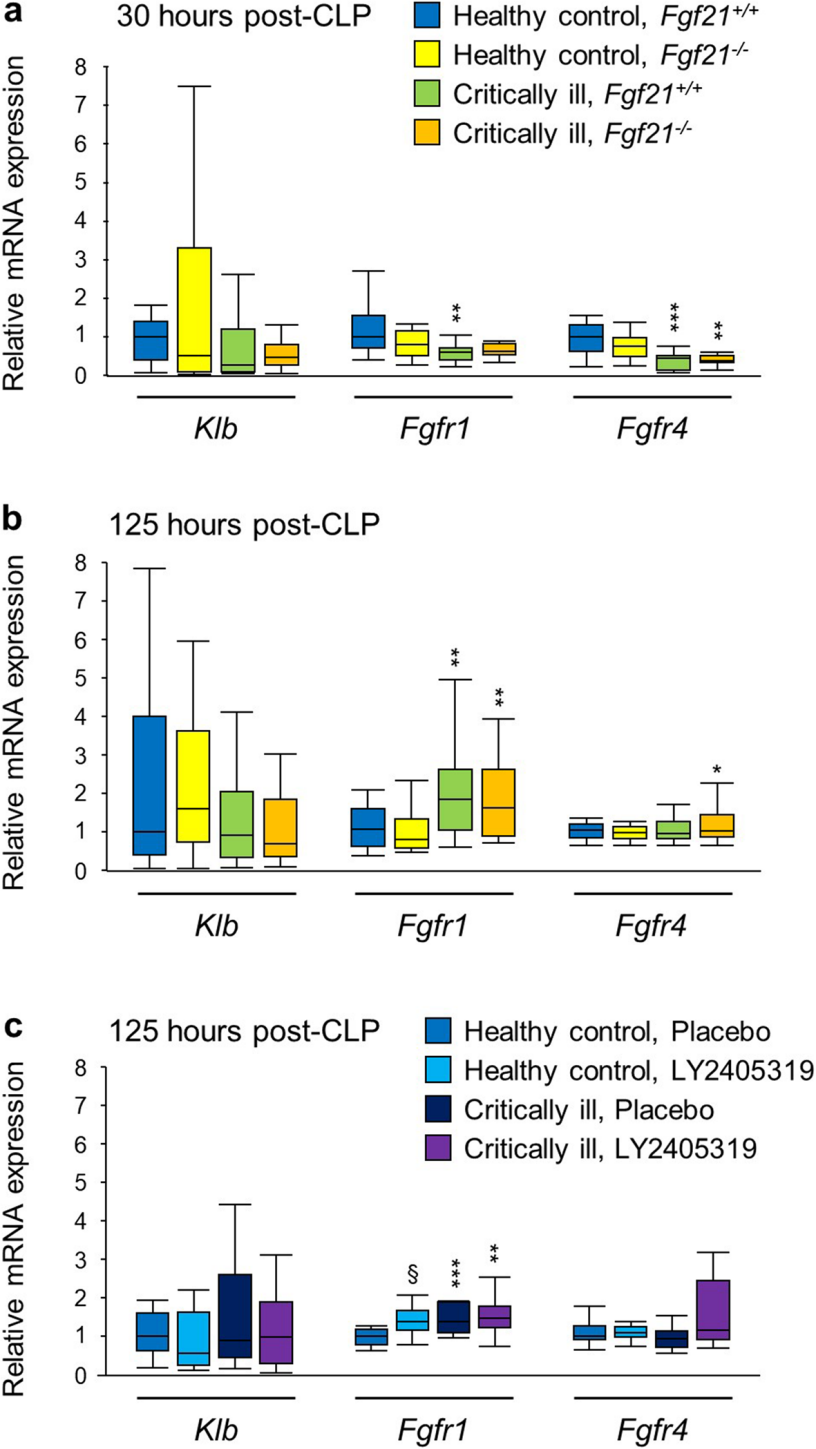
Effect of critical illness, and loss of FGF21 or FGF21 supplementation during critical illness on FGF21 receptor expression in muscle. Relative mRNA expression of *Klb*, *Fgfr1* and *Fgfr4* was quantified in gastrocnemius muscle, 30 h and 5 days after CLP, as compared with healthy pair-fed mice. **a** 30 h time point in the genetic FGF21 inactivation study. *n* = 15 for each group. **b** 5 days time point in the genetic FGF21 inactivation study. Healthy control, *Fgf21*^+*/*+^: *n* = 21; Healthy control, *Fgf21*^*−/−*^: *n* = 24; Critically ill, *Fgf21*^+*/*+^: *n* = 19; Critically ill, *Fgf21*.^*−/−*^: *n* = 18. **c** LY2405319 supplementation study. Healthy control, placebo: *n* = 18; Healthy control, LY2405319: *n* = 16; Critically ill, placebo: *n* = 16; Critically ill, LY2405319: *n* = 18. * *p* < 0.05, ** *p* < 0.01, *** *p* < 0.001 between healthy control and critically ill mice and § *p* < 0.05, §§ *p* < 0.01 between mice treated with placebo or with LY2405319

Given that effects of FGF21 loss or administration during critical illness were rather mild or absent, we investigated whether a degree of FGF21 resistance may play a role. Therefore, we quantified muscular gene expression of β-klotho (*Klb*, membrane-bound co-receptor required for FGF21 to bind and activate the fibroblast growth factor receptor (FGFR)), *Fgfr1* (highest affinity receptor) and *Fgfr4* (weaker affinity receptor not conveying signal transduction) [6]. Critical illness did not affect *Klb* expression at either time point (Fig. 5a,b). After 30 h, critical illness decreased expression of *Fgfr1* in *Fgf21*^+*/*+^ mice (*p* = 0.002) and of *Fgfr4* in both genotypes (*p* ≤ 0.001), whereas after 5 days, it increased expression of *Fgfr1* in both genotypes (*p* ≤ 0.005) and of *Fgfr4* in *Fgf21*^*−/−*^ mice (*p* < 0.05). Administration of LY2405319 increased *Fgfr1* expression in pair-fed healthy mice. Whereas critical illness induced its expression in placebo-treated mice, this was not the case in LY2405319-treated mice. The treatment did not affect expression of *Klb* or *Fgfr4* (Fig. 5c).

## Discussion

This study in a mouse model of critical illness induced by surgery and sepsis, in comparison with healthy pair-fed mice, confirmed that FGF21 rises acutely after the insult and remains elevated throughout the prolonged phase of illness. Genetic inactivation of FGF21 suggested that endogenous FGF21 is not necessary for survival, but may partially protect mice against critical illness-induced loss of body-weight and muscle force, possibly related to attenuation of ER stress. Unfortunately, however, exogenous supplementation with FGF21 to supraphysiological levels did not improve muscle force.

In this model of critical illness, plasma FGF21 acutely rose after the surgery and induction of sepsis, decreased towards the prolonged phase of illness, but remained elevated as compared with levels in healthy pair-fed mice, as in critically ill patients [20]. In these patients, higher FGF21 levels associated with an increased risk of death [20, 40]. Mortality was not the primary focus of our study but was documented to investigate potential bias in the molecular analyses. Neither loss of FGF21, nor FGF21 supplementation affected 5-day mortality in this clinically relevant model. This may seem in contrast with the protection against lipopolysaccharide (LPS)- or CLP-induced mortality with FGF21 supplementation to leptin-deficient female Ob/Ob mice (though not wildtype mice) or with the higher mortality observed in FGF21-deficient mice after LPS injection [22, 23]. Apart from genotype/animal strain and obese phenotype, several other differences may underly the apparently discrepant findings, including a higher baseline mortality of those sepsis models, female versus male sex, inadequate or no fluid resuscitation, a greater portion of the caecum being ligated and lack of antibiotic treatment.

Body-weight loss in prolonged critical illness was aggravated by loss of FGF21, but counter-acted by FGF21 supplementation. This suggests that the physiological rise in FGF21 protects against weight loss during critical illness. In the context of 24-h full fasting and nutrient restriction thereafter, FGF21 may have reduced energy expenditure, protecting mice from body-weight loss. This may be deduced from studies on fasting [41] or low protein, low-energy intake [42], conditions accompanied by an endogenous rise in FGF21 and decreased energy expenditure. Similarly, fasted FGF21 transgenic mice have decreased body temperature and locomotor activity compared to fasted wild-type mice [43]. In contrast, endogenous FGF21 increased energy expenditure in the setting of low protein, high-energy intake, contributing to weight loss [42, 44], and FGF21 overexpression or pharmacological administration of FGF21 had similar effects in the context of obesity or diabetes [45, 46].

Our study showed that endogenous FGF21 may partially protect muscle force in critical illness. *Fgf21*^*−/−*^ mice had a higher absolute muscle force and more larger myofibers under healthy pair-fed condition but a similar force and myofiber size distribution after induction of critical illness as compared with *Fgf21*^+*/*+^ mice. This suggests a greater critical illness-induced loss of muscle force and shift towards smaller myofibers in the absence of FGF21. Muscle weight, however, was equally better preserved in *Fgf21*^*−/−*^ than in *Fgf21*^+*/*+^ mice under healthy pair-fed and ill condition. Relative fasting may have played a role in the muscle mass preservation in *Fgf21*^*−/−*^ pair-fed healthy mice, as FGF21 has been implicated in fasting-induced loss of muscle mass, via attenuated protein synthesis and increased mitophagy flux [18]. In the present study, however, loss of FGF21 did not affect the protein synthesis-stimulating mTOR pathway, phosphorylation of eIF2α (suppressor of general protein synthesis in stress) or autophagy markers in muscle of healthy or critically ill mice. Nevertheless, differences in these dynamic pathways may not have been picked up by our static measurements in the absence of tracer or flux studies. Whether an effect on total number of myofibers, although mostly genetically determined [47], may explain the apparent discrepancy between effects on muscle weight versus myofiber size distribution, could not be determined. Functionally, we also did not find any difference in mitochondrial respiratory chain enzyme activities. However, loss of FGF21 increased ER stress. This effect may have contributed to the more pronounced loss of muscle force and increased E3 ligase expression, considering that ER stress has been associated with contractile dysfunction in myopathies and the UPR can induce these E3 ligases [48].

Because loss of FGF21 aggravated the illness-induced loss of muscle force, FGF21 supplementation could be protective. However, supraphysiological FGF21 supplementation mostly did not mirror the effects of FGF21 loss. Several explanations may be put forward. First, lack of a pronounced effect could theoretically be related to the use of a human FGF21 analogue, which was created for better stability [38]. However, this compound has previously shown biological activity in mice [38], which we confirmed with an ameliorated lipid profile and more weight loss in healthy mice. Second, it has been argued that FGF21 would not activate FGF21 signaling in muscle, due to lack of the obligatory β-klotho co-receptor [49]. However, several studies have confirmed *Klb* expression in muscle of both humans and mice, albeit at a very low level compared to liver [19, 49–56], in line with our finding. Furthermore, compelling evidence does point to effects of FGF21 on skeletal muscle [57], although in both beneficial and harmful directions. FGF21 administration promoted myogenic differentiation and enhanced mitochondrial function of C2C12 cells [58, 59], alleviated fluoride-induced atrophy of C2C12-derived myotubes [60], protected against hypoxia-reoxygenation-induced C2C12 cell injury [61] and potentiated glucose transport in mouse skeletal muscle fibers or human skeletal muscle-derived cultured myotubes [62, 63]. FGF21 overexpression alleviated skeletal muscle ischemia–reperfusion injury in mice [61], whereas FGF21 deficiency aggravated inflammation and atrophic responses in skeletal muscle of obese mice [64]. However, muscle FGF21 was also shown to be required for fasting-induced muscle atrophy and weakness (as mentioned above) [18] and has been implicated in ER stress-induced muscle catabolism [19]. Finally, another plausible explanation for lack of major effect of FGF21 supplementation on muscle in our study is that the already strongly increased endogenous FGF21 levels may have prevented additional effects from exogenous supplementation. Anyway, our study does not support the administration of FGF21 to attenuate muscle weakness in critically ill patients.

The potential protection of muscle force by endogenous FGF21 that we observed was at best mild and exogenous supplementation did not beneficially affect the muscle in a clinically relevant mouse model of critical illness. This contrasts with more pronounced protective effects observed in other organs in several other rodent models. For instance, FGF21 treatment alleviated LPS-induced acute lung injury [24, 25] and protected against acetaminophen- or D-galactose-induced acute liver injury and cisplatin-induced acute kidney injury [28–30]. FGF21 also showed brain-protective effects, with attenuation of LPS- or traumatic brain injury-induced behavior deficits [26, 65] and of ischemia-induced cerebral injury, motor disability, cognitive defects and ER stress [66, 67]. However, FGF21 treatment was started before or simultaneously with the organ insult, unlike the more clinically relevant delay of 10 h after CLP in our study.

Our study has some limitations. First, we studied the impact of loss of FGF21 in 16-weeks old mice instead of the 24-weeks age at which the model was developed and validated [33]. This may have impacted our findings, though was necessary to avoid chronic premorbid metabolic changes reported in standard chow-fed *Fgf21*^*−/−*^ mice [68]. Importantly, impact of critical illness on the studied markers was mostly comparable for both studies. Loss of FGF21 did affect some of the studied markers in healthy mice, but this was likely explained by the fasting/nutrient restriction [18] inherent to the pair-feeding to match the critical illness context. Nevertheless, pre-existing differences cannot completely be excluded as fully fed healthy mice as baseline were not studied. Second, we did not include sham-operated mice and did not administer antibiotics or pain medication to healthy pair-fed mice, as we did not aim to study the separate impact of sepsis, but rather the complete phenotype of critical illness caused by the complex interplay of disease and ICU treatments. Third, we studied static markers of autophagy, ER stress, protein synthesis and metabolism, but did not perform tracer or flux studies. Finally, although the animal model mimics several important characteristics of critically ill patients, caution is warranted with extrapolation to the human setting.

## Conclusions

Endogenous FGF21 is not required for surviving critical illness induced by surgery and polymicrobial sepsis in mice but may exert some mild adaptive, muscle-protective effects, with attenuation of muscle weakness and lowering of cellular stress in muscle. Supraphysiological FGF21 supplementation failed to improve muscle force. Thus, our study does not provide support for the use of FGF21 supplementation to improve muscular outcome of critically ill patients.

### Supplementary Information

**Additional file 1.** Compiled file with all additional information: Additional methods describing RNA isolation, reverse transcription, and real-time polymerase chain reaction; Protein isolation and immunoblotting; Mitochondrial enzyme activity; and Histological analyses; Additional tables describing Gene expression assays, Housekeeping gene Ct values, and Antibodies used for Western blot analyses; as well as Additional Figures illustrating Genotyping in liver, showing Example Western blots, and showing Mitochondrial complex activities expressed as citrate synthase ratio.

## Data Availability

Data sharing will be considered only on a collaborative basis with the principal investigators, after evaluation of the proposed study protocol.

## Abbreviations

ANOVA: Analysis of variance
*Aft4*: Activating transcription factor 4
ATF6: Activating transcription factor 6
*Calr*: Calreticulin
CLP: Caecal ligation and puncture
*Ddit3*: DNA Damage Inducible Transcript 3
EDL: Extensor digitorum longus
eIF2α: Eukaryotic translation initiation factor 2α
ER: Endoplasmic reticulum
*Fbxo32*: F-box protein 32 (also known as atrogin-1)
FGF21: Fibroblast growth factor 21
HEPES: 4-(2-Hydroxyethyl)-1-piperazineethanesulfonic acid
*Hspa5*: Heat shock protein family A (Hsp70) member 5
ICU: Intensive care unit
IRE1α: Inositol-requiring enzyme 1α
LC3-II: Microtubule-associated protein light chain-3, mature form
LPS: Lipopolysaccharide
mTOR: Mammalian target of rapamycin
p70S6K: P70S6 kinase (also known as ribosomal protein S6 kinase beta-1)
*Pdia4*: Protein disulfide isomerase family A member 4
PERK: PKR-like endoplasmic recticulum kinase
PN: Parenteral nutrition
*Trim63*: Tripartite motif containing 63 (also known as muscle ring finger 1)
UPR: Unfolded protein response
XBP1: X-box binding protein 1
*Xbp1s*: Spliced X-box binding protein 1
