# Supplementary material for: Development of muscle weakness in a mouse model of critical illness: does fibroblast growth factor 21 play a role?
Source: Skelet Muscle. 2023 Aug 4;13:12. doi: 10.1186/s13395-023-00320-4 (PMC10401744; doi:10.1186/s13395-023-00320-4)
Supplement: Supplementary file 1 — Additional file 1. Compiled file with all additional information: Additional methods describing RNA isolation, reverse transcription, and real-time polymerase chain reaction; Protein isolation and immunoblotting; Mitochondrial enzyme activity; and Histological analyses; Additional tables describing Gene expression assays, Housekeeping gene Ct values, and Antibodies used for Western blot analyses; as well as Additional Figures illustrating Genotyping in liver, showing Example Western blots, and showing Mitochondrial complex activities expressed as citrate synthase ratio. [file 13395_2023_320_MOESM1_ESM.docx]

**ADDITIONAL FILE 1**

**Development of muscle weakness in a mouse model of critical illness:**

**does FGF21 play a role?**

Wouter Vankrunkelsven,^1^ Steven Thiessen,^1^ Sarah Derde,^1^ Ellen Vervoort,^1^ Inge Derese,^1^ Isabel Pintelon,^2^ Hanne Matheussen,^1^ Alexander Jans,^1^ Chloë Goossens,^1^ Lies Langouche,^1^ Greet Van den Berghe,^1,#^ Ilse Vanhorebeek^1,#^

^1^ Clinical Division and Laboratory of Intensive Care Medicine, Department of Cellular and Molecular Medicine, KU Leuven, Leuven, Belgium.

^2^ Laboratory of Cell Biology and Histology, University of Antwerp, Antwerp, Belgium

# Equally contributed

**Corresponding author**: Ilse Vanhorebeek, MEng, PhD Laboratory of Intensive Care Medicine, KU Leuven, Herestraat 49, B-3000 Leuven, Belgium; Tel +32 16 330532; Fax +32 16 344015; email ilse.vanhorebeek@kuleuven.be ; ORCID ID: 0000-0002-5261-5192.

**Table of contents**

**Methods S1:** RNA isolation, reverse transcription, and real-time polymerase chain reaction

**Methods S2:** Protein isolation and immunoblotting

**Methods S3:** Mitochondrial enzyme activity

**Methods S4:** Histochemical analyses

**Table S1:** Gene expression assays

**Table S2:** Ct values of Rn18S as housekeeping gene

**Table S3:** Antibodies used for Western blot analyses

**Figure S1:** Illustration of liver genotyping of *Fgf21^+/+^* and *Fgf21^-/-^* mice

**Figure S2:** Illustration of Western blots on muscle of healthy and critically ill *Fgf21^+/+^* and *Fgf21^-/-^* mice

**Figure S3:** Effect of loss of FGF21 on muscle mitochondrial function during critical illness

**Figure S4:** Effect of LY2405319 supplementation on muscle mitochondrial function during critical illness

**Methods S1: RNA isolation, reverse transcription, and real-time polymerase chain reaction**

RNA was isolated with Qiazol and the RNeasy mini RNA isolation kit (QIAGEN, Venlo, The Netherlands). DNAse treatment removed genomic DNA. RNA was reverse-transcribed with the use of random hexamers. Relative gene expression was determined with the 2^-ΔΔCt^ method with 18S ribosomal RNA (*Rn18s*) as housekeeping gene. An overview of the assays used for gene expression is provided in Table S1.

**Methods S2: Protein isolation and immunoblotting**

Tissue samples were homogenised in a buffer containing 20 mM Tris-HCl, pH 7.6, 10% glycerol, 1% Nonidet P-40, 2 μg/ml aprotinin, 5 μg/ml leupeptin, 0.5 μg/ml pepstatin, 10 mM sodium orthovanadate, 34 μg/ml phenylmethylsulfonyl fluoride, 10 mM sodium pyrophosphate, 100 mM sodium fluoride, and 10 mM EDTA. The protein content was determined with Coomassie Protein Assay Reagent (Pierce Biotechnology Inc.) using a standard curve of BSA. Western blots were performed using commercial 4-20% tris-glycine gels (Biorad, Hercules, CA) and PVDF membranes (Thermo Fisher scientific, Waltham, MA). A list of the primary antibodies used and the respective dilution is shown in Table S3. Secondary horseradish peroxidase-conjugated antibodies were purchased from DakoCytomation (Heverlee, Belgium). Blots were developed with the Western Lightning chemiluminescence reagent Plus kit (Perkin Elmer, Zaventem, Belgium), visualised with the G:BOX Chemi XRQ (SynGene, Cambridge, UK) and analyzed with the SynGene software.

**Methods S3: Mitochondrial enzyme activity**

Citrate synthase and mitochondrial respiratory chain complex activities were measured with spectrophotometry at 30°C as described previously [1]. Tibialis anterior muscle (whole muscle) was homogenised in homogenisation buffer (210 mM mannitol, 70 mM sucrose, 5 mM HEPES, 1 mM EGTA, pH 7.2) on ice with a Dounce homogeniser, followed by five freeze-thaw cycles in liquid nitrogen. An additional sonication step was performed for measurements of citrate synthase and complex I activity. Activities were calculated by subtracting the blank measurements from the total absorbance. Samples were measured in triplicate.

Citrate synthase activity was measured in a buffer containing 50 mM KPi, pH 7.0 and 100 µM DTNB. The reaction was started by adding the homogenate, 100 µM acetyl-CoA and 100 µM oxaloacetic acid, pH 7.2. For blanks, oxaloacetic acid was omitted. The production of the thionitrobenzoate anion was followed at 412 nm.

Complex I activity was measured in a buffer containing 50 mM KPi pH 7.4, 50 µM NADH, 1 mM KCN, 10 µM antimycin A, 1 mg/ml BSA and 50 µM coenzyme Q1. The reaction was started after adding the homogenate. To the blank, the complex I inhibitor rotenone (2.5 µM) was added. NADH consumption was followed at 340 nm.

Complex V activity was measured in a Tris-bicarbonate buffer (40 mM Tris-HCO3, 1 mM EGTA, pH 8.0) to which 200 µM NADH, 2.5 mM PEP, 5 µM antimycin A, 5 mM MgCl2, 27.5 U/ml lactate dehydrogenase, and 10 U/ml pyruvate kinase were added. After a pre-incubation step with 2.5 mM ATP, homogenate was added to start the reaction. For measurements in muscle, two blanks were included for which either ATP or homogenate was omitted from the mixture. For liver samples, oligomycin (2 µmol/l) was added to the blank reaction. The consumption of NADH was followed at 340 nm.

**Methods S4:** **Histochemical analyses**

Tibialis anterior muscle samples were fixed in paraformaldehyde and embedded in paraffin. Subsequent stainings were performed on 5-7 µm cross-sectional sections.

To evaluate general structure, 5 µm muscle sections were stained with hematoxylin and eosin. The sections were scored for presence of angular fibers, adipocyte infiltration, fibrosis and signs of inflammation.

To quantify myofiber size distribution, a laminin immunohistochemical staining was performed on freshly cut 7 µm sections. After deparaffination and washing 5 minutes with distilled water, antigen retrieval was performed with Target Retrieval Solution (S1699, Dako, Glostrup, Denmark) for 1 hour at 95°C, followed by 20 minutes cooling down. Sections were washed 3 times (one rinsing and twice 5 minutes incubation) in Tris-buffered saline (TBS) with 0.025% Triton X-100. Sections were then blocked with 10% normal goat serum (X0907, Dako) in TBS with 1% bovine serum albumin (A7906, Sigma-Aldrich, Saint Louis, MO, USA) for 30 minutes at room temperature, followed by overnight incubation at 4°C with anti-laminin antibody (ab11575, Abcam, Cambridge, UK, 1:50 in phosphate buffered saline (PBS) with 1% normal goat serum). After washing 3 times 5 minutes in PBS, sections were incubated for 1 hour at 37°C with goat-anti-rabbit-AF488 (A11008, Invitrogen, Glasgow, UK, 1:500 in PBS with 1% normal goat serum). Finally, sections were again washed 3 times 5 minutes in PBS and were mounted in Aqua-Poly/Mount (18606-100, Polysciences, Warrington, PA, USA). Whole slides were scanned with a TissueFAXS i PLUS microscope (TissueGnostics, Vienna, Austria). Myofiber segmentation was performed with the deep learning algorithm “Cellpose” [2]. Quantification of the myofibers was performed with use of the “LabelsToRoi” plugin in ImageJ, which allows processing Cellpose generated label images [3]. The plugin overlays the Cellpose-generated segmentations with the raw file followed by automatic quantification of the cross-sectional area of each myofiber. The segmentation erosion tool function within this plugin, designed to counter staining-dependent area bias was set at 1 after optimisation with visual inspection. Finally, with use of JMP Pro 17.0.0 (SAS Institute Inc, Cary, NC), cross-sectional areas of the myofibers for each of the samples were subsequently classified to bins of 200 µm^2^, followed by generation of smoothed curves of the myofiber size distribution for each of the experimental groups.

**Table S1: Gene expression assays**

| **Gene symbol** | **TaqMan Assay ID (Applied biosystems)** |
| --- | --- |
| *Atf4* | Mm00515325_g1 |
| *Calr* | Mm00482936_m1 |
| *Ddit3* | Mm01135937_g1 |
| *Fbxo32* | Mm00499523_m1 |
| *Fgf21* | Mm00840165_g1 |
| *Hspa5* | Mm00517690_g1 |
| *p62* | Mm00448091_m1 |
| *Pdia4* | Mm00437958_m1 |
| *Rn18s* | Mm03928990_g1 |
| *Trim63* | Mm01185221_m1 |
| **Gene symbol** | **Primer and probe sequences (Eurogentec)** |
| *Xbp1s* | Forward primer: 5’-CTG AGT CCG CAG CAG GT-3’ (900 nM)  Probe: 5’-GGC CCA GTT GTC ACC TCC CC-3’ (300 nM)  Reverse primer: 5’-TGT CAG AGT CCA TGG GAA GA-3’ (900 nM) |

**Table S2: Ct values of Rn18S as housekeeping gene**

|  | **Ct *Rn18s*** | **P-value** |
| --- | --- | --- |
| **Day 1 FGF21 knockout study** |  | 0.65 |
| Healthy control, *Fgf21*^+/+^ | 11.63 (11.39 – 12.04) |  |
| Healthy control, *Fgf21*^-/-^ | 11.56 (11.35 – 11.97) |  |
| Critically ill, *Fgf21*^+/+^ | 11.66 (11.50 – 11.93) |  |
| Critically ill, *Fgf21*^-/-^ | 11.62 (11.22 – 11.78) |  |
| **Day 5 FGF21 knockout study** |  | 0.13 |
| Healthy control, *Fgf21*^+/+^ | 9.04 (8.89 – 9.13) |  |
| Healthy control, *Fgf21*^-/-^ | 9.04 (8.80 – 9.33) |  |
| Critically ill, *Fgf21*^+/+^ | 9.19 (8.77 – 9.44) |  |
| Critically ill, *Fgf21*^-/-^ | 9.23 (8.94 – 9.48) |  |
| **Day 5 FGF21 supplementation study** |  | 0.70 |
| Healthy control, placebo | 8.55 (8.24 – 8.63) |  |
| Healthy control, LY2405319 | 8.50 (8.21 – 8.73) |  |
| Critically ill, placebo | 8.46 (8.06 – 8.60) |  |
| Critically ill, LY2405319 | 8.55 (8.32 – 8.71) |  |

**Table S3: Antibodies used for Western blot analyses**

| **Antibody** | **Source** | **Product number** | **Dilution** |
| --- | --- | --- | --- |
| Anti-LC3B | Sigma-Aldrich | L7543 | 1/1000 TBST-5% BSA |
| Anti-p62 | Novus Biologicals | H00008878-M01 | 1/1000 TBST-5% BSA |
| Anti-eIF2α | Cell Signaling Technology | #5324 | 1/1000 TBST-5% BSA |
| Anti-eIF2αS1 | Abcam | Ab32157 | 1/1000 TBST-5% BSA |
| Anti-p70 S6 Kinase | Cell Signaling | #9202 | 1/1000 TBST-5% BSA |
| Anti Phospho-p70 S6 Kinase | Cell Signaling | #9205 | 1/1000 TBST-5% BSA |

**Figure S1: Illustration of liver genotyping of *Fgf21^+/+^* and *Fgf21^-/-^* mice**

SeqF1/F21GRev6-1 primers amplify a 500 bp segment of *Fgf21^+/+^* DNA and SeqF1/NeoRev3 primers amplify a 300 bp segment of *Fgf21^-/-^* DNA. Red numbers reflect the sizes of the fragments in the loaded DNA ladder.

**Figure S2:** **Illustration of Western blots on muscle of healthy and critically ill *Fgf21^+/+^* and *Fgf21^-/-^* mice**

Marks in the center of the Western blot images indicate molecular weight markers in kDa.

**Figure S3:** **Effect of loss of FGF21 on muscle mitochondrial function during critical illness**

Respiratory chain complex I and V activity in tibialis anterior muscle are expressed relative to the activity of citrate synthase (CS) as mitochondrial marker enzyme. Healthy control, *Fgf21^+/+^*: n=21; Healthy control, *Fgf21^-/-^*: n=24; Critically ill, *Fgf21^+/+^*: n=19; Critically ill, *Fgf21^-/-^*: n=18. * p<0.05 between healthy control and critically ill mice.

**Figure S4:** **Effect of LY2405319 supplementation on muscle mitochondrial function during critical illness**

Respiratory chain complex I and V activity in tibialis anterior muscle are expressed relative to the activity of citrate synthase (CS) as mitochondrial marker enzyme. Healthy control, placebo: n=18; Healthy control, LY2405319: n=16; Critically ill, placebo: n=16; Critically ill, LY2405319: n=18. * p<0.05 between healthy control and critically ill mice.

**References**

1. Vanhorebeek, I., De Vos, R., Mesotten, D., et al., 2005. Protection of hepatocyte mitochondrial ultrastructure and function by strict blood glucose control with insulin in critically ill patients. Lancet. 365, 53-59. doi:10.1016/S0140-6736(04)17665-4.

2. Stringer C, Wang T, Michaelos M, Pachitariu M. Cellpose: a generalist algorithm for cellular segmentation. Nat Methods. 2021;18:100-106. doi: 10.1038/s41592-020-01018-x.

3. Waisman A, Norris AM, Elías Costa M, Kopinke D. Automatic and unbiased segmentation and quantification of myofibers in skeletal muscle. Sci Rep. 2021;11(1):11793. doi: 10.1038/s41598-021-91191-6.
